# Relationship of Post-Transplant Lymphoproliferative Disorders (PTLD) Subtypes and Clinical Outcome in Pediatric Heart Transplant Recipients: A Retrospective Single Institutional Analysis/Experience of 558 Patients

**DOI:** 10.3390/cancers15030976

**Published:** 2023-02-03

**Authors:** Yan Liu, Billy C. Wang, Craig W. Zuppan, Peter Chau, James Fitts, Richard Chinnock, Jun Wang

**Affiliations:** 1 Department of Pathology and Laboratory Medicine, Loma Linda University School of Medicine, Loma Linda, CA 92354, USA; 2 Division of Pediatric Critical Care, Department of Pediatrics, University of Illinois College of Medicine Peoria, Peoria, IL 61605, USA; 3 Department of Cancer Biology and Pharmacology, University of Illinois College of Medicine Peoria, Peoria, IL 61605, USA; 4 Division of Pediatric Cardiology, Department of Pediatrics, University of California San Diego, San Diego, CA 92123, USA; 5 Department of Pediatrics, Loma Linda University School of Medicine, Loma Linda, CA 92354, USA

**Keywords:** post-transplant, lymphoproliferative disorder, heart transplant, lymphoma, EBV, CMV, survival

## Abstract

**Simple Summary:**

Post-transplant lymphoproliferative disorders (PTLD) are heterogenous lymphoproliferative disorders that develop in immunosuppressed transplant recipients. We performed a retrospective review of PTLD occurring in pediatric heart transplant recipients and sought to determine the correlation of PTLD subtypes with different characteristics. Our single institution retrospective study found that compared to older children, infant heart transplant recipients were less likely to develop PTLD. Infant heart transplant recipients who developed PTLD were diagnosed later than older children and had a lower rate of more aggressive PTLD. The overall survival of patients with more aggressive PTLD was significantly lower than patients with low-grade PTLD. Proper classification of the type of PTLD is important, as the subtypes of PTLD showed a significant correlation with the outcome.

**Abstract:**

Post-transplant lymphoproliferative disorders (PTLD) are heterogenous lymphoproliferative disorders that develop as a consequence of immunosuppression in transplant recipients. We sought to determine if subtypes of PTLD correlated with different outcomes. We performed a retrospective review of PTLD occurring in pediatric heart transplant recipients. A total of 558 children and infants underwent cardiac transplantation at our institution between 1985 and 2019 and were followed until March 2021. Forty-nine of 558 patients developed PTLD (8.8%). As compared to older children (>one year of age), infant recipients (<three months of age) were less likely to develop PTLD. Monomorphic PTLDs (M-PTLD, 61%) was the most common subtype at initial diagnosis, followed by non-destructive (21%), polymorphic (14%), and classic Hodgkin lymphoma (cHL, 4%). Patients who underwent transplantation at a young age (<three months) had significantly lower rates of M-PTLD or cHL and a longer time from transplant to PTLD diagnosis as compared to children older than one year at transplant (*p* = 0.04). Although not reaching statistical significance, patients with a shorter time to PTLD diagnosis showed a trend toward higher rates of M-PTLD or cHL. As expected, the overall survival (OS) of patients with M-PTLD or cHL was significantly lower than patients with non-destructive or polymorphic PTLD.

## 1. Introduction

Post-transplant lymphoproliferative disorders (PTLD) represent a spectrum of lesions ranging from infectious mononucleosis and lymphoid/plasmacytic hyperplasia to aggressive, malignant lymphoid neoplasms that occur in adult or pediatric immunosuppressed patients following solid organ or hematopoietic stem cell (HSC) transplantation [1,2,3]. These lesions are usually associated with Epstein–Barr Virus (EBV) infection in the setting of an immunosuppression [4,5,6]. Accurate diagnosis requires histopathology, immunophenotype, detection of EBV-encoded RNA (EBER), and other studies. Based on the 2017 revision of the World Health Organization (WHO) classification and as continuously recognized by the most recent International Consensus Classification (2022 ICC) of PTLD, there are four categories: non-destructive (ND), polymorphic (P), monomorphic (M), and classic Hodgkin lymphoma (cHL) [2,3]. While previous studies have characterized risk factors for disease development [7,8,9,10,11], few have characterized the clinicopathologic correlation of PTLD and its subtypes.

Our institution has extensive experience with pediatric heart transplantation; the first successful neonatal heart transplant occurred in our institution in 1985, and it currently has performed more pediatric heart transplants than any other center with over 30 years of long-term follow-up [12,13,14] Here, we report our single institution experience with clinical characters and disease outcomes in PTLD and its subtypes in pediatric heart transplant recipients. Our findings may provide prognostic, treatment, and outcome guidance for transplant providers and patients.

## 2. Materials and Methods

Our institutional files were searched for all cases of PTLD occurring in pediatric patients (<18 years) who underwent cardiac transplantation between 1985 and September 2019. These were reviewed and re-classified according to the 2017 WHO categorization [3] and as continuously recognized by the most recent 2022 international consensus classification (2022 ICC) of the mature lymphoid neoplasms [2]. Patient follow-up was evaluated through March 2021. The subtypes of PTLD diagnoses were further analyzed with respect to several clinical parameters.

Clinical data gathered included gender, race, age at transplantation, time from transplantation to development of PTLD, location and classification of the lesion(s), donor and recipient CMV/EBV status at the time of transplantation, recipient EBV infection status after transplant, donor and recipient blood and Rh type, overall survival ((OS), time from PTLD development to death). If PTLD was discovered on autopsy, time from PTLD development to death was classified as “zero days”. Survival time was calculated from the date of pathologic diagnosis to the date of death or last encounter.

Kaplan–Meier survival curves were used to estimate the overall survival and analyzed by the log-rank method. Fisher’s exact test, unpaired *t*-test, and chi-square test were used to compare PTLD distribution by different groups. A *p* < 0.05 was used for statistical significance. Statistical analyses were performed using GraphPad Prism 9.1.1.

This retrospective study was approved by the LLUMC Institutional Review Board (IRB Approval # 53306).

## 3. Results

### 3.1. General Clinical Features

**Figure 1:**
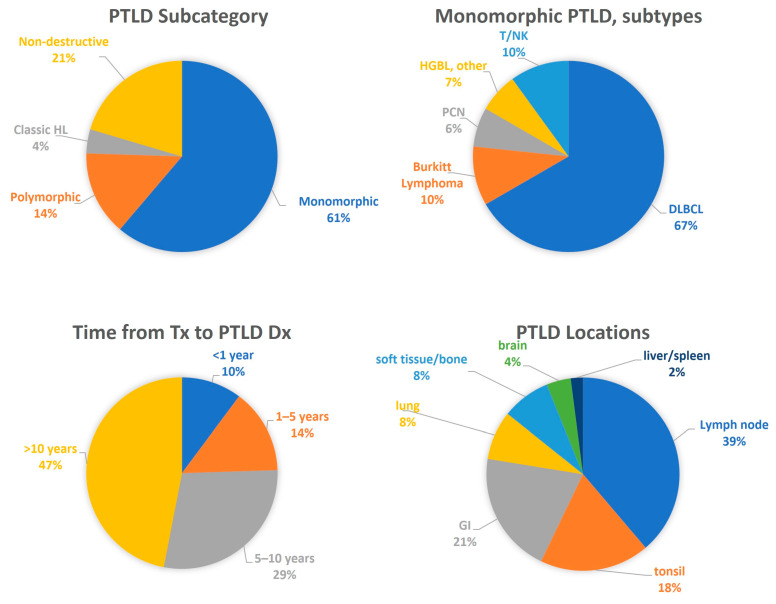
General clinical features in PTLD patients.

A total of 558 pediatric patients underwent heart transplantation at our institution between 1985 and September 2019, including 348 infants (<one year) and 210 older children (>1–18 years). Of these, 49 (8.8%) were diagnosed with PTLD during the study period (31 males and 18 females). PTLD was diagnosed in 30 of 348 infants (8.6%) and 19 of 210 older children (9.0%). In five patients, the PTLD either progressed or recurred as a higher grade over time. Among the 49 patients with PTLD, 22 had undergone transplantation in the first three months of life. Time from transplant to development of PTLD ranged from 6 months to 21.9 years, with a median of 9.6 years and 47% occurring more than 10 years after transplantation (Figure 1). In those patients who died, the median overall survival following diagnosis was 0.74 years, and 60.8% of deaths were directly attributable to PTLD.

Monomorphic PTLD (61%) was the most common category at initial diagnosis, followed by non-destructive (21%), polymorphic (14%), and cHL (4%) (Figure 1). Among M-PTLDs, diffuse large B-cell lymphoma (DLBCL) was the most common subtype (67%) (Figure 1). Within ND-PTLDs, the infectious mononucleosis (IM)-like subtype was most common (70%). The primary sites of PTLD included lymph node (39%), tonsil (18%), gastrointestinal tract (21%), lung (8%), soft tissue/bone (8%), brain (4%), and liver/spleen (2%). All 4 cases of plasmacytic lesions (plasmacytic hyperplasia (2) and plasma cell neoplasm (2)) were in Hispanic/Latino patients.

**Figure 2:**
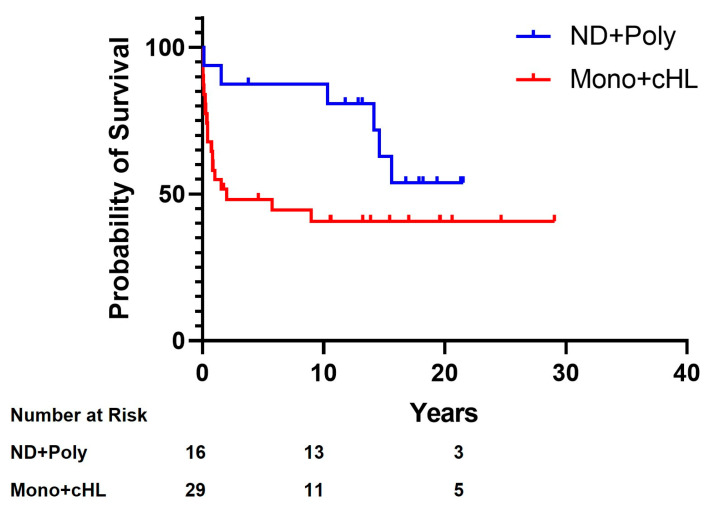
Overall survival by PTLD subtypes. ND: Non-destructive; Poly: Polymorphic; Mono: Monomorphic (*p* = 0.021).

As might be expected, given their more aggressive phenotype, the M-PTLD and cHL group showed lower overall survival compared to the ND-PTLD and P-PTLD group (*p* = 0.021, Figure 2) and lower five-year OS (48.8% vs. 86.8%).

These results are summarized in Table 1 and graphically represented by Figure 1 and Figure 2.

### 3.2. Effect of Age at Transplant

**Figure 3:**
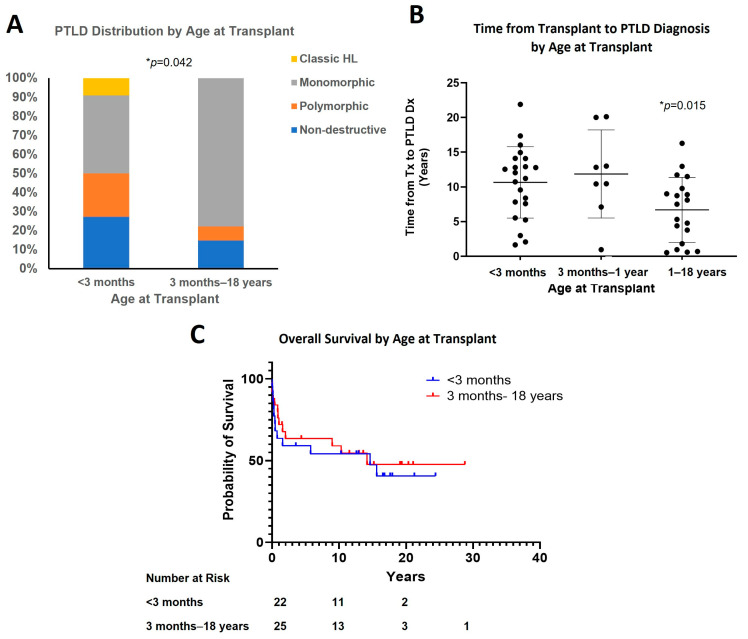
Effect of age at transplant. (**A**) PTLD distribution by age at transplant; (**B**) Time from transplant to PTLD diagnosis by age at transplant (*p* = 0.015, <3 months vs. 1–18 years); (**C**) Overall survival by age at transplant.

Aggressive forms of PTLD (M-PTLD and cHL) comprised a smaller proportion of the PTLD arising in patients transplanted under three months of age, as compared to those who were older (>three months) when they were transplanted (50% vs. 78%) (Figure 3A and Table 1, *p* = 0.042). Patients who were younger at the time of transplant had a longer post-transplant interval before developing PTLD as compared to older children with a mean of 10.7 years and 6.7 years, respectively (for <three months vs. one year to 18 years, *p* = 0.015) (Figure 3B). There were eight patients who underwent transplant between three months to one year of age, and these showed a frequency of M-PTLD (75%) similar to older children (Figure S1A) but a time from transplant to PTLD diagnosis similar to the younger (<three months) group (11.9 years) (Figure 3B).

No statistically significant difference was observed in the survival of patients among different subgroups (Figure 3C and Figure S1C). Patients younger than three months at the time of transplant had similar median survival to those who were three months or older (14.7 years vs. 14.2 years), while median survival in the subgroup of one year or older at transplant was shorter (10.3 years). Similar five-year overall survival was observed in patients younger than three months and one year or older at transplant (57.1% vs. 58.8%). The five-year overall survival in the three-month to the one-year group was 66.7%, but there was insufficient data to separately analyze median survival.

### 3.3. Effect of Time Interval from Transplant to PTLD Diagnosis

**Figure 4:**
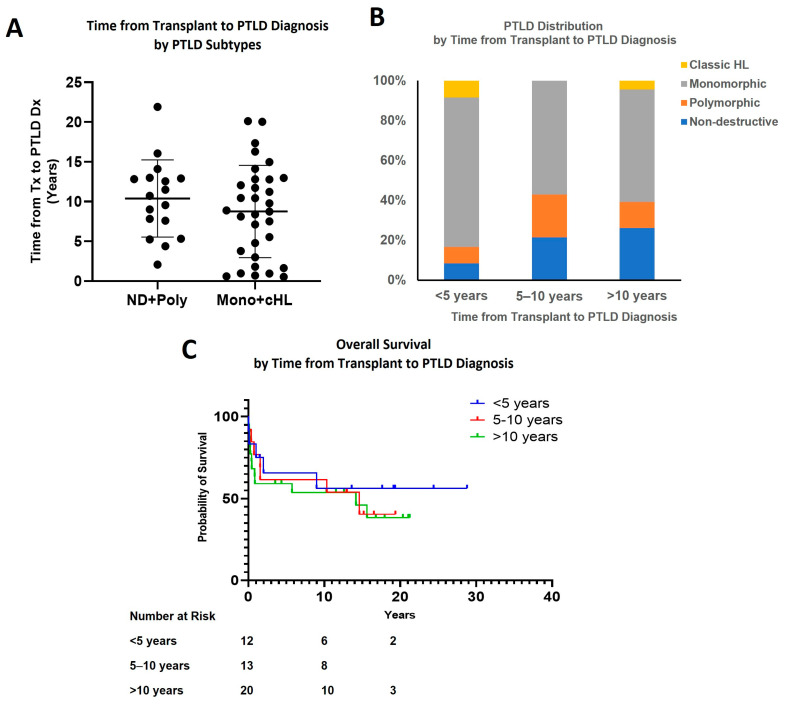
Effect of time interval from transplant to PTLD diagnosis. (**A**) Time from transplant to PTLD diagnosis by PTLD subtype; (**B**) PTLD distribution by time from transplant to PTLD diagnosis; (**C**) Overall survival by time from transplant to PTLD diagnosis.

There was a trend toward more aggressive PTLD associated with a shorter interval from transplantation to PTLD, but this did not reach statistical significance (Figure 4A). The incidence of M-PTLD and cHL was highest in patients who developed PTLD within five years of transplant (10 of 12, 83.3%), compared to 57% in those who developed it 5 to 10 years after the transplant and 60.9% in those who developed it more than 10 years after the transplant (Figure 4B and Table 1). All five patients who developed PTLD within one year after transplant developed M-PTLD. There was no statistically significant difference in survival among groups based on the time interval from transplant to PTLD diagnosis (Figure 4C). The five-year OS was 63.5% in the <five-year group, 61.5% in the 5–10-year group, and 55% in the >10-year group.

### 3.4. EBER and EBV Infection Status

As EBV was not routinely tested in the early era of transplantation at our institution, only 16 patients who developed PTLD were tested for EBV status at the time of transplantation. Among the five known EBV-negative recipients, two received their hearts from EBV-positive donors, and in the remaining three, the EBV status of the donor was unknown. All five EBV-negative recipients developed M-PTLD, with three DLBCL (EBER positive), one high-grade B-cell lymphoma (HGBL), and one plasma cell neoplasm. Eight of the 11 EBV-positive recipients developed M-PTLD, with five DLBCL and three Burkitt lymphomas.

EBER testing was performed on 42 of the 49 PTLD lesions and was positive in most of the cases (79%, *n* = 33/42). Subtype analysis revealed EBER positivity as follows: ND-PTLD (*n* = 8/8, 100%); P-PTLD (*n* = 5/6, 83%); M-PTLD (*n* = 18/26, 69%); and cHL-PTLD (*n* = 2/2, 100%). Plasma EBV DNA and/or antibody results were available in 36 out of 49 cases, with only one EBV infection-negative case (DLBCL, M-PTLD). Discordance was observed between tumor EBER status and EBV infection status in three cases, in which the tumor was EBER-negative while the patient had an EBV infection. These were two cases of Burkitt lymphoma and one case of DLBCL.

Tumor EBER status and EBV infection status are summarized in Table 1.

### 3.5. Effect of CMV Status

**Figure 5:**
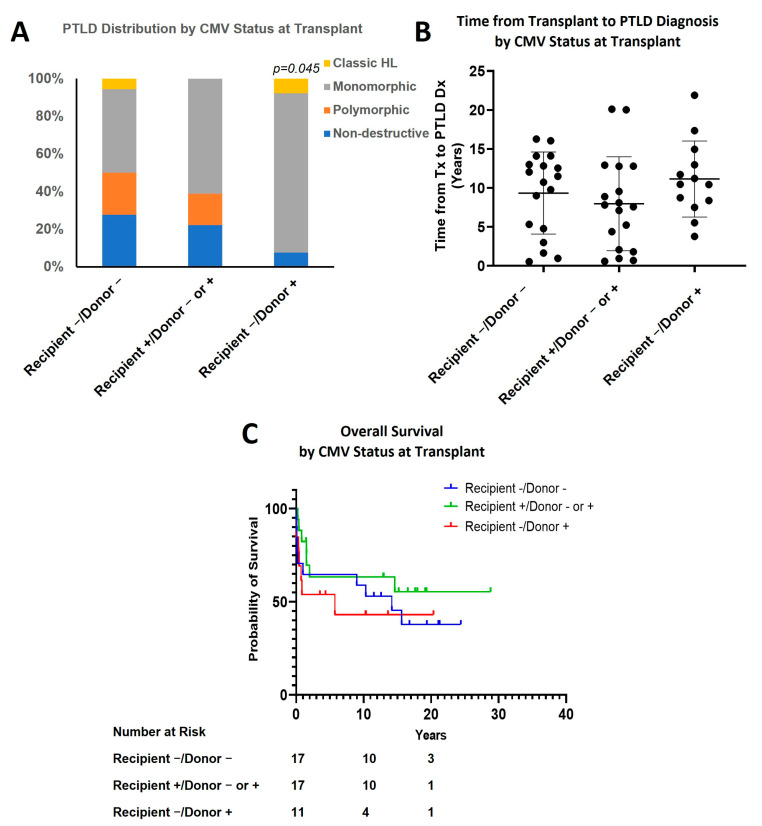
Effect of CMV status. (**A**) PTLD distribution by CMV status at transplant (*p* = 0.045, recipient−/donor+ vs. combined other two groups); (**B**) Time from transplant to PTLD diagnosis by CMV status at transplant; (**C**) Overall survival by CMV status at transplant.

CMV-naïve recipients who received a transplant from CMV positive donor (recipient−/donor+) had a higher incidence of M-PTLD or cHL subtypes (92.3%) than the remaining patients (recipient−/donor−: 50%; recipient+/donor+ or −: 61.1%; *p* = 0.045; Figure 5A and Table 1). However, there was no statistically significant difference in the time interval from transplant to PTLD diagnosis (recipient−/donor−: 9.3 ± 5.3 years (mean ± SD); recipient+/donor+ or −:8.0 ± 6.0; recipient−/donor+: 11.2 ± 4.9; Figure 5B). The recipient-/donor+ group had a lower median survival (5.7 years) and five-year OS (45.5%) compared to the other two groups, but this was not statistically significant (15.6 years, 63.6%, combined two groups; Figure 5C).

### 3.6. Effect of Recipient and Donor Blood Type

There were 12 recipients who received transplants from donors with either ABO or Rh mismatch. There was no significant difference in PTLD type, time from transplant to development of PTLD, or survival, as compared to those without mismatch (Figure S2 and Table 1).

## 4. Discussion

PTLD is a potentially life-threatening complication of solid-organ transplantation. The risk of developing PTLD is reported to be highest within the first year following transplantation and decreases thereafter [15,16,17]. Risk factors include EBV infection, the degree of immunosuppression, the type of organ transplanted, and the age at the time of transplant surgery [18,19,20,21]. The prognosis is associated with age at PTLD diagnosis, recipient’s EBV status, bone marrow involvement, and best initial response [8,22]. In this single-institution retrospective study, we identified patients who developed PTLD after heart transplantation and analyzed histologic and clinical characteristics.

In comparison to another single-institution study of PTLD-complicating transplants of all types [8], our patients had a considerably longer time interval from transplant to PTLD diagnosis (median time 9.6 years) with only 10% of PTLD occurring within the first year, and 25% within five years. In our cohort, there was a longer interval from transplant to PTLD diagnosis in younger recipients (<three months of age) than in old recipients (>one year of age). This finding is consistent with that reported in other studies [7,23,24]. The overall longer time interval from transplant to PTLD diagnosis in our patients, as compared to all transplant patients, may be related to the predominance of infants in our cohort. Immunosuppression regimens at the time of induction and maintenance differ based on patient factors, including age. Infants who are non-sensitized typically receive lower immunosuppression in our institution, which may affect the development and type of PTLD (i.e., infants < three months had a less “aggressive” type of PTLD). Furthermore, there may be a protective effect of maternal antibodies against EBV [25].

Our study also showed a trend of the more aggressive subtypes of PTLD (M-PTLD or cHL) to associate, on average, with a shorter time interval to the development of PTLD (83% vs. 60% at <five years vs. >five years). M-PTLD/cHL cases occurred slightly earlier than ND/polymorphic -PTLD cases (mean 8.8 years vs. 10.4 years). Although these data are intriguing and suggest a correlation between the PTLD subtype and the time interval from transplant to PLTD diagnosis, these differences did not reach statistical significance, and additional study in this area is needed.

ND-PTLD tends to regress with the reduction in immune suppression, and the prognosis is usually excellent, particularly in children [26,27]. Polymorphic-PTLD may also regress with the reduction in the immune suppression [28,29]. The combined M-PTLD/cHL group showed significantly lower overall survival compared to the ND/P-PTLD group in our study. However, the recipients < three months of age (who had a lower proportion of M-PTLD or cHL) did not show a statistically significant difference in overall survival compared to other groups. This suggests that other patient factors, in combination with PTLD subtypes, may contribute to the outcome in infants.

In our study, the CMV mismatch group (seropositive donor with the seronegative recipient) had both a higher rate of developing M-PTLD or cHL and a lower five-year overall survival and median overall survival compared to those in which CMV status was matched or the recipient was seropositive (45.5% vs. 63.6%). While Epstein–Barr virus (EBV) infection is a clearly known risk factor for the development of PTLD, the role of CMV infection has been controversial [18,30,31]. In one study, the increased risk of PTLD with CMV mismatch was found to be related to concomitant EBV mismatch. Although CMV was not an independent risk or prognostic factor for PTLD, it appeared to enhance the effects of the EBV [30]. As EBV status was not routinely tested in the early era of transplant at our institution, the EBV mismatch status is unknown in most of our cases, which precluded analyzing for the separate and concomitant effects of EBV and CMV mismatch.

As a retrospective single-institution study, the relatively small size of the cohort might limit the significance of our findings to a certain extent. In addition, the limitation of complete and thorough data collection may also compromise the design of our study on confounding factors. The long timespan of this study may also cause potential bias due to differential losses of follow-up. As a result, more definite conclusions may be drawn on larger cohort studies in the future.

## 5. Conclusions

In summary, our study indicates that pediatric heart transplant recipients develop the full spectrum of PTLD, as seen in their adult counterparts. We found that patients who received their heart transplant at the age younger than three months had a longer time interval to the development of PTLD and a lower rate of more aggressive subtypes (M-PTLD or cHL) as compared to recipients older than one year of age, although this did not translate into a significant difference in overall survival. Although not statistically significant, we also demonstrated a trend toward a shorter time interval from transplant to PTLD diagnosis in patients with M-PTLD or cHL. Our study further suggests that CMV mismatch may be associated with a higher rate of developing M-PTL or cHL. Overall, our findings suggest that subtyping PTLD in pediatric heart transplant recipients may assist in prognostication and support the potential need to alter PTLD maintenance screening protocols based on patient risk factors.

## Figures and Tables

**Table 1:** Clinical characteristics and subtypes of PTLD.

	Total	ND-PTLD			P-PTLD	M-PTLD					cHL-PTLD
		PH	IM-Like	FFH		DLBCL	Burkitt	HGBL, Other	PCN	T/NK	
All	49	2	7	1	7	20	3	2	2	3	2
Genders											
Male	31	1	4	0	4	13	2	0	2	3	2
Female	18	1	3	1	3	7	1	2	0	0	0
Race											
Caucasian	22	0	5	1	4	8	0	1	0	2	1
Hispanic/Latino	21	2	2	0	2	9	2	1	2	1	0
Others	6	0	0	0	1	3	1	0	0	0	1
Tumor EBER status											
Positive	33	1	6	1	5	13	1	1	1	2	2
Negative	9	0	0	0	1	4	2	0	1	1	0
Not available	7	1	1	0	1	3	0	1	0	0	0
EBV infection											
Positive	35	1	7	0	6	15	3	1	1	1	0
Negative	1	0	0	0	0	1	0	0	0	0	0
Not avaible	13	1	0	1	1	4	0	1	1	2	2
Tumor EBER status/EBV infection											
Pos/Pos	28	1	6	0	5	12	1	1	1	1	0
Neg/Neg	1	0	0	0	0	1	0	0	0	0	0
Neg/Pos	3	0	0	0	0	1	2	0	0	0	0
Age at Tx											
<3 months	22	0	6	0	5	5	1	0	1	2	2
3 months–18 years	27	2	1	1	2	15	2	2	1	1	0
Time from Tx to PTLD Dx											
<1 year	5	0	0	0	0	3	0	1	1	0	0
1–5 years	7	1	0	0	1	3	1	0	0	0	1
5–10 years	14	1	2	0	3	6	2	0	0	0	0
>10 years	23	0	5	1	3	8	0	1	1	3	1
CMV status at Tx											
Recipient−/Donor−	18	1	3	1	4	3	2	0	2	1	1
Recipient−/Donor+	13	0	1	0	0	9	1	0	0	1	1
Recipient+/Donor− or +	18	1	3	0	3	8	0	2	0	1	0
Blood type and Rh status											
Recipient O+/Donor O+ or O− or Recipient B+/Donor B+ or B− or Recipient A+/Donor A+ or A−	37	2	7	1	5	16	2	1	1	1	1
Recipient A+ or B+/Donor O+	7	0	0	0	1	2	0	1	1	2	0
Recipient A−/Donor A+ or Recipient O−/Donor O+	5	0	0	0	1	2	1	0	0	0	1

ND: non-destructive; PTLD: post-transplant lymphoproliferative disorder; P-PTLD: polymorphic-PTLD; M-PTLD: monomorphic-PTLD; PH: plasmacytic hyperplasia; IM: infectious mononucleosis; FFH: florid follicular hyperplasia; DLBCL: diffuse large B-cell lymphoma; HGBCL: high-grade B-cell lymphoma; PCN: plasma cell neoplasm; T/NK: T-cell or NK-cell lymphoma; cHL: classic Hodgkin lymphoma; Tx: transplantation; Dx: diagnosis.

## Data Availability

The data presented in this study are available upon request from the authors.

## Supplementary Materials

The following supporting information can be downloaded at: https://www.mdpi.com/article/10.3390/cancers15030976/s1; Figure S1: Effect of age at transplant; Figure S2: Effect of recipient and donor blood type.
