# Supplementary material for: Relationship of Post-Transplant Lymphoproliferative Disorders (PTLD) Subtypes and Clinical Outcome in Pediatric Heart Transplant Recipients: A Retrospective Single Institutional Analysis/Experience of 558 Patients
Source: Cancers (Basel). 2023 Feb 3;15(3):976. doi: 10.3390/cancers15030976 (PMC9913467; doi:10.3390/cancers15030976)
Supplement: Supplementary file 1 [file cancers-15-00976-s001.zip › cancers-2097876-supplementary.pdf]

cancers

MDPI

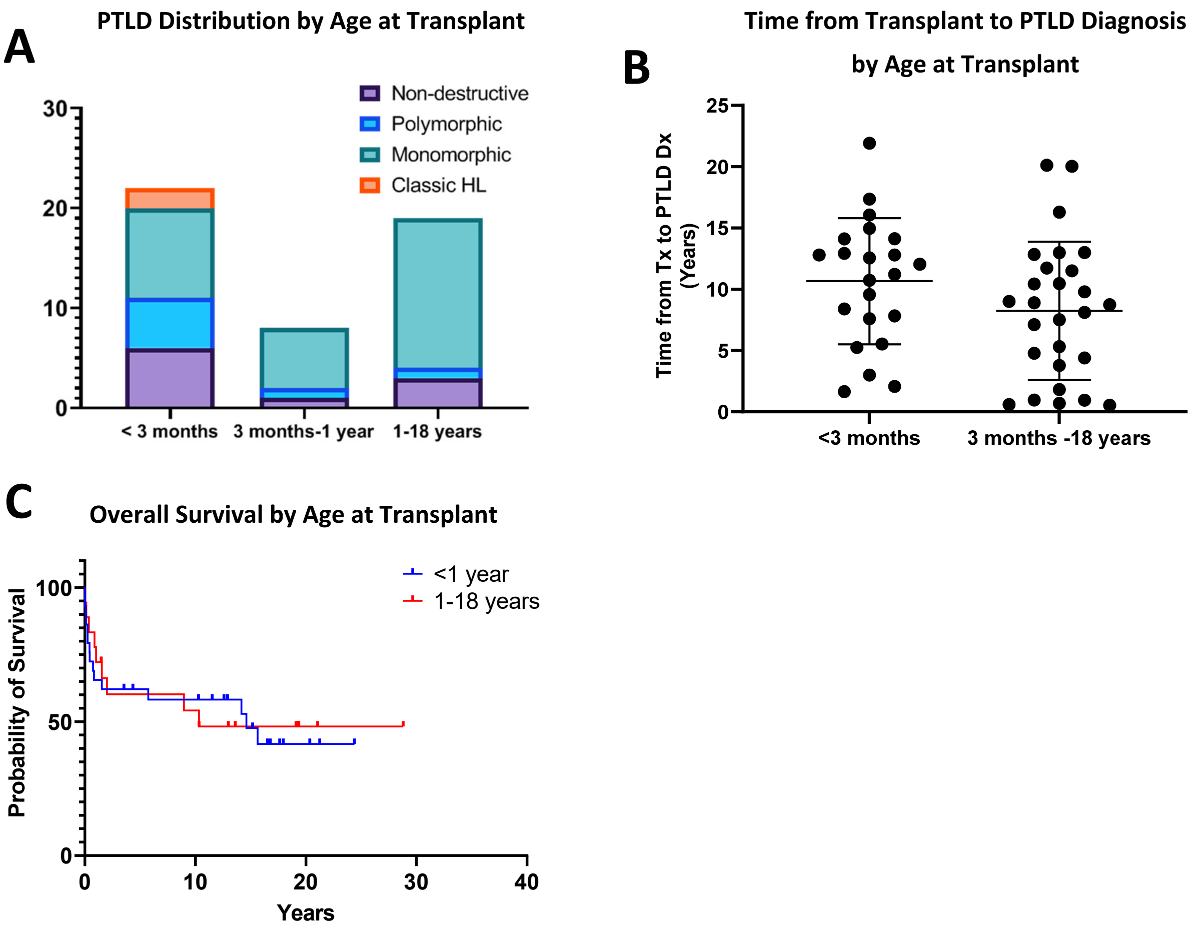


**Figure S1. Effect of age at transplant.** (**A**) PTLD distribution by age at transplant. (**B**) Time from transplant to PTLD diagnosis by age at transplant. (**C**) Overall survival by age at transplant.

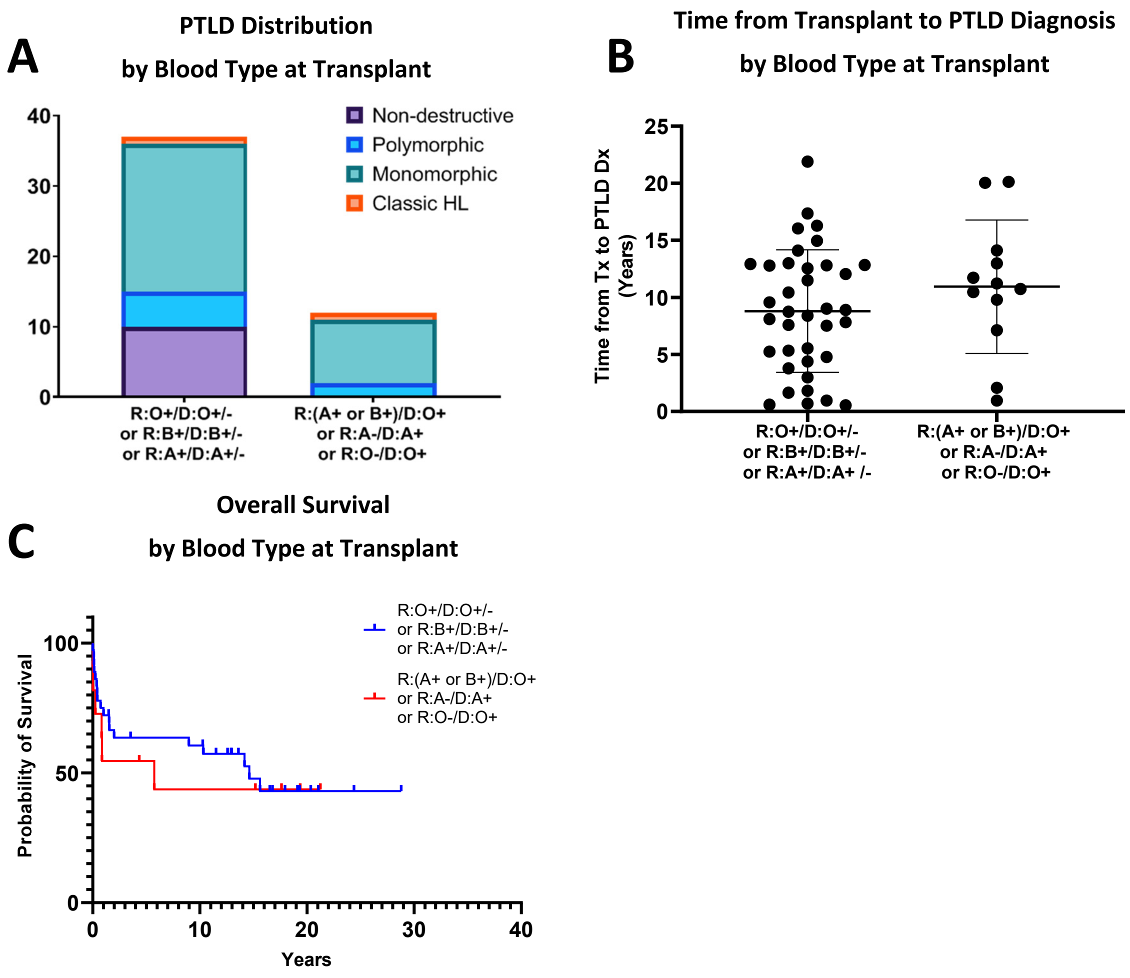


**Figure S2. Effect of recipient and donor blood type.** (**A**) PTLD distribution by blood type at transplant. (**B**) Time from transplant to PTLD diagnosis by blood type at transplant. (**C**) Overall survival by blood type at transplant.
